# Non-occlusive mesenteric ischemia complicated by ischemic hepatitis presenting as severe lactic acidosis: a case report

**DOI:** 10.3389/fmed.2026.1909717

**Published:** 2026-07-20

**Authors:** Huan Geng, Hui Ye, Ming Lei

**Affiliations:** Department of Critical Care Medicine, Seventh People’s Hospital of Shanghai University of Traditional Chinese Medicine, Shanghai, China

**Keywords:** case report, continuous renal replacement therapy, ischemic hepatitis, lactic acidosis, non-occlusive mesenteric ischemia

## Abstract

**Background:**

Non-occlusive mesenteric ischemia (NOMI) and ischemic hepatitis (IH) are severe target-organ injuries caused by splanchnic hypoperfusion and are associated with substantial mortality, particularly in elderly patients with panvascular disease. When intestinal and hepatic hypoperfusion occur concurrently, intestinal anaerobic metabolism may increase lactate production, whereas ischemic hepatocellular injury may impair lactate clearance. Together, these mechanisms can lead to severe or even refractory lactic acidosis.

**Case presentation:**

An 84-year-old man was admitted for progressive black discoloration of the skin on his left foot lasting more than 1 month. His medical history included hypertension, type 2 diabetes mellitus, cerebral infarction, lower-extremity arteriosclerosis obliterans, prior lower-limb endovascular intervention, and left femoral artery stent implantation. He had been receiving long-term dual antiplatelet therapy (DAPT). On postoperative day 10 after debridement and skin grafting of the left foot, he suddenly developed coffee-ground emesis, followed by tachypnea and lethargy. Although blood pressure remained relatively stable during this episode, he developed tachycardia, mottled skin, severe anemia, metabolic acidosis, and marked hyperlactatemia, with lactate levels exceeding 20 mmol/L. After transfer to the intensive care unit (ICU), fasting, fluid resuscitation, blood transfusion, acid–base correction, and continuous renal replacement therapy (CRRT) were initiated, with gradual improvement in acid–base balance and metabolic status. Contrast-enhanced abdominal computed tomography (CT) revealed extensive atherosclerosis of the abdominal aorta and its branches, mural thrombi, penetrating atherosclerotic ulcers, and ischemic changes in the bowel and right hepatic lobe. After multidisciplinary evaluation, the patient was diagnosed with NOMI complicated by IH. Following comprehensive intensive treatment, his condition stabilized, and he was discharged successfully.

**Conclusion:**

NOMI should be considered in elderly patients with panvascular disease and perioperative volume fluctuations who present with unexplained hyperlactatemia and metabolic acidosis. Clinicians should recognize that splanchnic hypoperfusion may affect multiple target organs simultaneously. Early recognition and timely multidisciplinary management may help improve outcomes in this high-risk population.

## 1 Introduction

Lactic acidosis is a common metabolic disturbance in critically ill patients and is associated with poor prognosis. Clinically, it is generally defined as a persistently elevated arterial lactate level (>5 mmol/L) accompanied by a pH < 7.35 (1, 2). In perioperative and intensive care settings, tissue hypoperfusion, an imbalance between oxygen supply and demand, and impaired lactate clearance are major mechanisms underlying lactate elevation (2–7). Serial lactate measurements may therefore provide an early indication of inadequate tissue perfusion.

Acute mesenteric ischemia (AMI) refers to a group of disorders caused by an acute reduction or interruption of mesenteric blood flow, which may lead to intestinal ischemia and, in severe cases, bowel necrosis. Although AMI accounts for only 0.09%–0.20% of emergency admissions, its mortality rate remains as high as 50%–80% (4–10). Non-occlusive mesenteric ischemia (NOMI) accounts for approximately 20%–30% of all AMI cases (9, 11). Unlike arterial embolism or thrombosis, NOMI is not primarily caused by mechanical arterial occlusion but rather by redistribution of splanchnic blood flow and persistent mesenteric vasospasm during systemic hypoperfusion (12–14). It commonly occurs in elderly, critically ill, or medically complex patients. Because early symptoms are non-specific and typical abdominal pain may be absent, diagnosis is often delayed (4, 9, 11).

Ischemic hepatitis (IH), also known as hypoxic hepatitis, is a diffuse hepatocellular injury caused by an abrupt reduction in effective hepatic perfusion. It is typically characterized by a rapid and marked increase in serum aminotransferase levels and is associated with an in-hospital mortality rate of 45%–73% (15–17). When NOMI coexists with IH, intestinal ischemia increases lactate production, whereas ischemic hepatocellular injury reduces lactate clearance. The combined effect of these two mechanisms may result in severe or even refractory lactic acidosis (18).

Cases of imaging-confirmed NOMI complicated by IH presenting predominantly with severe lactic acidosis have rarely been reported. In accordance with the CARE case report guidelines (19), we report the diagnosis and management of an 84-year-old patient with panvascular disease who developed NOMI complicated by IH after foot debridement and skin grafting. This case highlights the clinical value of serial lactate monitoring and early imaging evaluation in high-risk perioperative patients.

## 2 Case presentation

An 84-year-old man was admitted on 28 March 2026, with a more than 1-month history of progressive dark discoloration of the skin on his left foot. His medical history included hypertension, type 2 diabetes mellitus, cerebral infarction, lower-extremity arteriosclerosis obliterans, bilateral lower-extremity balloon angioplasty, and left femoral artery stent implantation. He had been receiving amlodipine besylate for blood pressure control and subcutaneous insulin for glycemic control. He was also on long-term dual antiplatelet therapy with enteric-coated aspirin and clopidogrel.

After admission, the patient underwent debridement and autologous skin grafting of the left foot on 31 March and 9 April, respectively, with satisfactory postoperative wound healing. On 19 April, the patient developed sudden vomiting of approximately 50 mL of coffee-ground gastric contents without visible clots, subsequently accompanied by tachypnea and lethargy. High-flow humidified oxygen therapy (FiO2 70%) was promptly initiated. Physical examination showed a heart rate of 115 beats/min, respiratory rate of 32 breaths/min, blood pressure of 126/68 mmHg, peripheral oxygen saturation of 96%, and scattered skin mottling. Abdominal examination revealed a soft abdomen without tenderness, rebound tenderness, or guarding, with decreased bowel sounds. Laboratory tests and arterial blood gas analysis revealed the following: pH 7.21, PaO_2_ 344.1 mmHg, PaCO_2_ 23.7 mmHg, HCO_3_^–^ 9.3 mmol/L, lactate 18.7 mmol/L, base excess −17.07 mmol/L, hemoglobin 58 g/L, alanine aminotransferase 13.8 U/L, serum creatinine 147.2 μmol/L, and a positive fecal occult blood test. Bedside ultrasonography showed relatively preserved cardiac systolic function and a narrow, collapsed inferior vena cava. Given the findings of effective circulating volume depletion, severe anemia, tissue hypoperfusion, and severe metabolic acidosis, the patient was transferred to the ICU for further management.

After ICU admission, fasting, acid suppression, and gastric protection with esomeprazole sodium, fluid resuscitation with sodium lactate Ringer’s solution, transfusion of packed red blood cells and fresh frozen plasma, and correction of acidosis with sodium bicarbonate were promptly initiated. However, metabolic acidosis continued to worsen, and lactate increased beyond the upper detection limit (>20 mmol/L). Because refractory lactic acidosis was suspected, CRRT was initiated to correct the severe acid–base disturbance and stabilize metabolic status (Table 1). During treatment, liver enzyme levels progressively increased, with ALT rising from 122.1 to 1237.5 U/L and peaking at 1768.6 U/L, suggesting possible acute ischemic liver injury. Hepatoprotective therapy with reduced glutathione for injection and monoammonium glycyrrhizinate and cysteine hydrochloride sodium chloride injection was administered accordingly (Table 2).

**TABLE 1:** Arterial blood gas parameters and lactate levels at key time points.

Date	pH	PaCO_2_ (mmHg)	PaO_2_ (mmHg)	FiO_2_ (%)	Glucose (mmol/L)	BE (mmol/L)	HCO_3_ (mmol/L)	Lactate (mmol/L)
2026-04-19	7.210	23.7	344.1	70	16.4	−17.07	9.3	18.7
2026-04-19	7.080	13.8	407.7	50	7.1	−24.05	4.1	>20.0
2026-04-19	7.417	21.3	108.5	30	3.9	−9.21	16.5	>20.0
2026-04-19	7.407	35.4	NA	30	7.1	−2.81	22.3	19.8
2026-04-20	7.520	35.7	NA	30	7.5	6.19	28.9	8.2
2026-04-20	7.461	45.2	NA	30	7.1	6.91	30.2	5.5
2026-04-20	7.367	37.7	108.3	30	9.8	−3.43	21.2	3.2
2026-04-20	7.338	34.6	130.2	30	7.9	−6.62	18.8	3.1
2026-04-21	7.378	30.0	107.6	30	11.8	−6.52	18.8	4.2
2026-04-21	7.386	26.7	141.3	30	11.1	−7.75	17.8	1.9
2026-04-22	7.382	31.2	127.0	25	15.2	−5.71	19.4	1.6
2026-04-23	7.437	32.8	123.9	25	14.0	−1.62	22.6	1.6
2026-04-24	7.493	27.6	NA	25	10.5	−1.24	22.8	2.1
2026-04-25	7.477	27.3	93.2	25	8.9	−2.24	22.0	1.2

BE, base excess; CRRT, continuous renal replacement therapy; HCO_3_, bicarbonate; PaCo_2_, partial pressure of arterial carbon dioxide; PaO_2_, partial pressure of arterial oxygen. NA indicates unavailable data. Lactate levels exceeding the upper detection limit were recorded as >20.0 mmol/L.

**TABLE 2:** Serial laboratory findings during clinical deterioration and recovery.

Date and time	WBC (× 109/L)	RBC (× 1,012/L)	PLT (× 109/L)	Hb (g/L)	CRP (mg/L)	PT (s)	APTT (s)	D-dimer (mg/L)	ALT (U/L)	Scr (pmol/L)	cTnl (ng/ml)
2026-03-29 07:30	8.96	3.58	332	111	4.07	11.8	32.1	2.69	28.8	78.0	<0.01
2026-04-11 07:30	8.60	2.51	211	80	<0.5	11.8	29.5	1.75	14.4	75.7	NA
2026-04-19 07:30	10.88	1.83	227	58	<0.5	15	29.2	1.82	13.8	147.2	0.03
2026-04-19 14:30	19.46	1.96	251	63	3.85	NA	NA	NA	122.1	NA	NA
2026-04-19 21:30	16.52	1.74	239	56	NA	NA	NA	2.23	1237.5	102.1	NA
2026-04-20 07:30	7.25	2.67	125	84	30.5	25.5	42.7	8.26	1768.6	75.6	0.22
2026-04-21 07:30	18.11	3.18	124	99	158.85	30.3	57.9	9.00	NA	66.7	0.16
2026-04-22 07:30	21.94	2.88	123	93	158.89	16.9	49.6	4.86	1496.1	152.6	0.36
2026-04-23 07:30	14.40	2.93	101	94	79.3	13	42.4	6.19	828.9	104.5	0.31
2026-04-26 07:30	10.70	2.70	140	85	81.44	13.6	45.5	8.82	142.5	87.9	0.05
2026-04-29 07:30	12.62	2.47	215	75	54.11	13.1	44.8	4.18	45.7	87.4	0.01
2026-05-01 07:30	10.87	2.51	225	80	46.46	12.5	44.1	2.72	37.5	85.8	0.03
2026-05-04 07:30	5.19	2.23	208	NA	25.44	13.2	44.4	NA	32.4	89.6	0.01
2026-05-06 07:30	5.85	2.70	228	85	35.23	NA	NA	NA	NA	NA	NA
2026-05-10 07:30	10.92	2.96	349	90	117	12.6	44.4	3.73	17.1	119.1	0.01

ALT, alanine aminotransferase; APTT, activated partial thromboplastin time; CRP, C-reactive protein; cTnl, cardiac troponin I; D-dimer, D-dimer; Hb, hemoglobin; PLT, platelet count; PT, prothrombin time; RBC, red blood cell count; Scr, serum creatinine; WBC, white blood cell count. NA indicates unavailable data. Values preceded by “<” indicate results below the lower limit of detection.

**FIGURE 1:**
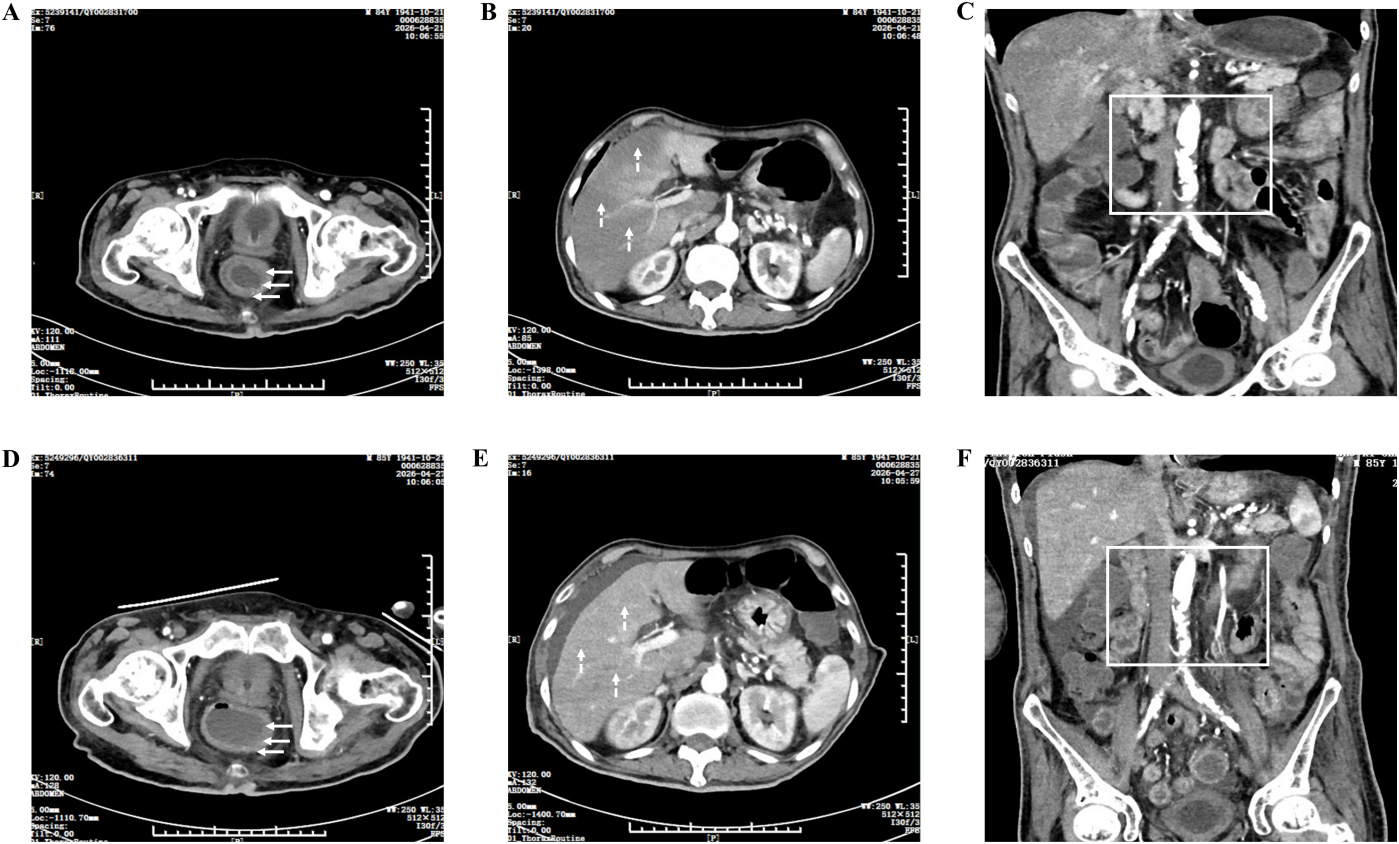
Contrast-enhanced abdominal computed tomography (CT) findings before and after treatment, showing changes in ischemic bowel injury, hepatic hypoperfusion, and penetrating atherosclerotic ulcer of the abdominal aorta. **(A,D)** Axial contrast-enhanced abdominal CT images before and after treatment, respectively, showing bowel changes. Before treatment, thickening and edema of the rectal/sigmoid colon wall were observed; these findings were alleviated after treatment. Solid white arrows indicate the affected bowel wall. **(B,E)** Axial contrast-enhanced abdominal CT images before and after treatment, respectively, showing hepatic perfusion changes. Before treatment, multiple patchy hypoenhancing areas were observed in the liver parenchyma, suggesting hepatic hypoperfusion/ischemia; hepatic enhancement improved after treatment. Dashed white arrows indicate the areas of hypoperfusion. **(C,F)** Coronal contrast-enhanced CT images before and after treatment, respectively, showing abdominal aortic lesions. A focal contrast-filled outpouching was observed in the abdominal aortic wall, consistent with mural thrombus and formation of a penetrating atherosclerotic ulcer. The lesion remained morphologically stable after treatment, without obvious progression. The white boxes indicate the PAU region, located in the infrarenal abdominal aorta.

On 21 April the patient’s vital signs had stabilized, and acid–base and electrolyte disturbances had improved. CRRT was discontinued, and contrast-enhanced abdominal CT was performed. The findings showed extensive atherosclerosis of the abdominal aorta and its branches, with mural thrombi and penetrating atherosclerotic ulcers. Variable degrees of luminal stenosis were observed in different vascular segments, along with ischemic changes in the bowel and right hepatic lobe (Figures 1A–C). The patient was ultimately diagnosed with NOMI complicated by IH. Treatment was then adjusted to include papaverine for relief of vasospasm, alprostadil to improve microcirculation, and piperacillin–tazobactam for infection control. Hepatoprotective therapy was continued, and parenteral nutrition was provided with medium- and long-chain triglyceride fat emulsion, amino acids, and glucose. Because gastrointestinal decompression yielded no obvious drainage, stool color had turned yellow-brown, and the hemoglobin level had stabilized, low-molecular-weight heparin anticoagulation was initiated after evaluation. Enoxaparin sodium 4000 anti-Xa IU was administered subcutaneously once daily. Blood counts, liver and kidney function, coagulation parameters, and stool characteristics were closely monitored.

**FIGURE 2:**
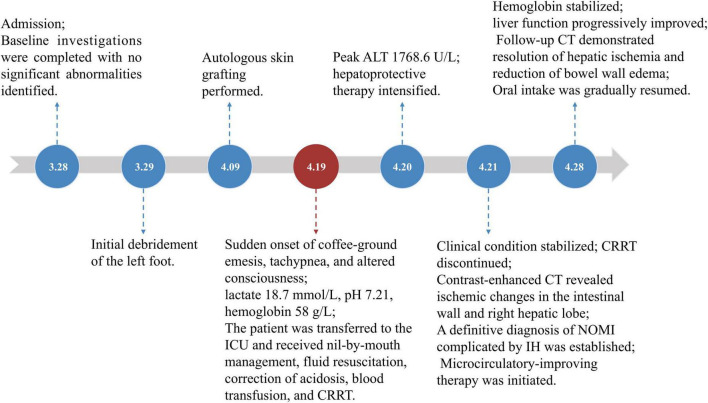
Timeline of key clinical events during hospitalization.

On 28 April the patient had no abdominal tenderness; his stool remained yellow-brown, hemoglobin levels were stable, and liver function had gradually improved. Follow-up abdominal CT showed alleviation of ischemia in the right hepatic lobe and reduced bowel wall edema (Figures 1D–F). Thereafter, oral intake was gradually resumed, beginning with water and progressing to a liquid diet, semi-liquid diet, and then a normal diet. The patient was discharged after his condition stabilized (Figure 2). At the 1-month follow-up after discharge, he had no abdominal pain, abdominal distension, melena, or other related symptoms. The clinical outcome was favorable.

## 3 Discussion

This case suggests that elderly patients with panvascular disease may develop severe splanchnic hypoperfusion after perioperative volume fluctuations. Even when systemic blood pressure appears stable, such patients may still develop NOMI because of limited vascular reserve and impaired microcirculatory compensation, with ischemic injury potentially involving multiple target organs. Notably, this patient had no typical abdominal pain or signs of peritoneal irritation in the early stage, and refractory lactic acidosis was the predominant manifestation. In high-risk perioperative patients, persistent lactate elevation should be regarded as an important warning sign of visceral ischemia rather than being attributed solely to postoperative stress or a systemic inflammatory response.

### 3.1 Pathophysiological link between panvascular disease and splanchnic hypoperfusion

The central pathophysiological process in this case was splanchnic hypoperfusion caused by severe underlying vascular disease combined with volume imbalance. The patient had a history of extensive atherosclerosis involving the cerebral, lower-extremity, and microvascular beds. Contrast-enhanced abdominal CT further confirmed extensive atherosclerosis of the abdominal aorta and its branches, with mural thrombi, indicating markedly limited splanchnic vascular reserve. Previous studies have shown that asymptomatic mesenteric arterial stenosis can be detected in 27%–40% of patients with peripheral arterial disease (11). In this setting, surgical trauma, potential bleeding, and stress responses may all reduce effective circulating volume and impair tissue oxygen delivery.

When effective circulating volume falls below an individual patient’s compensatory threshold, the renin–angiotensin–aldosterone system and sympathetic nervous system are activated to maintain perfusion of vital organs such as the heart and brain, resulting in sustained splanchnic vasoconstriction and redistribution of blood flow (14). This compensatory response may preserve systemic blood pressure to some extent, but at the expense of further mesenteric hypoperfusion. Previous studies have shown that severe or persistent intestinal ischemia can cause irreversible mucosal injury and even intestinal necrosis within a short time (9–11, 20–22). Once the intestinal mucosal barrier is disrupted, bacterial translocation and endotoxemia may further induce systemic inflammatory responses, thereby aggravating local intestinal injury and distant organ dysfunction (23).

Notably, a mild elevation in cardiac troponin I (cTnI) was observed during the clinical course. At the time of deterioration, the patient had multiple risk factors, including tachycardia, severe lactic acidosis, and effective circulating volume depletion. However, bedside ultrasonography showed preserved cardiac systolic function and a small, collapsed inferior vena cava. In addition, there was no typical chest pain, dynamic electrocardiographic changes, or regional wall motion abnormality to support a diagnosis of acute coronary syndrome. Taken together, the mild increase in cTnI was considered more consistent with secondary myocardial injury in the setting of systemic multiorgan hypoperfusion, rather than a primary cardiac event precipitating the hemodynamic disturbance. This interpretation was further supported by the subsequent clinical course: as lactate was gradually cleared and the internal milieu stabilized, cTnI decreased in parallel and returned to the normal range (Table 2), suggesting a clear temporal association. Nevertheless, the elevation in cTnI indicates substantial myocardial vulnerability during severe systemic hypoperfusion in such patients, warranting close attention, continuous electrocardiographic monitoring, and follow-up assessment of cardiac function.

### 3.2 The gut–liver dual hit in the development of severe lactic acidosis

Concurrent involvement of the intestine and liver was a key mechanism underlying the development of severe lactic acidosis in this case. NOMI caused extensive intestinal ischemia and enhanced anaerobic metabolism, thereby markedly increasing lactate production. In parallel, reduced portal venous return and hepatic arterial hypoperfusion jointly caused hypoxic hepatocellular injury, manifesting as IH (17, 18, 20). Because the liver is the major organ responsible for lactate metabolism and clearance (18), hepatocellular injury substantially impairs lactate clearance. Thus, this patient had both increased lactate production and impaired lactate clearance, ultimately leading to rapidly progressive, treatment-refractory lactic acidosis.

Based on serial laboratory data, lactate levels rose rapidly during the early phase of clinical deterioration and exceeded the upper limit of detection, whereas alanine aminotransferase (ALT) increased subsequently, peaking at 1768.6 U/L on 20 April. This temporal pattern suggests that the early hyperlactatemia was primarily driven by increased lactate production due to tissue hypoperfusion, while impaired lactate clearance secondary to ischemic hepatitis further contributed to and sustained the elevated lactate levels. It should be noted, however, that because lactate production rates and intestinal perfusion were not directly monitored, the relative contributions of increased lactate production and reduced lactate clearance cannot be quantified. Therefore, this interpretation remains a reasonable inference based on the clinical temporal sequence and requires validation in larger prospective studies.

In addition, long-term DAPT may have further complicated the clinical course. Under ischemic conditions, the intestinal mucosa is prone to micro-ulceration and focal necrosis. When platelet aggregation is inhibited, mucosal injury may more readily progress to gastrointestinal bleeding (24). Bleeding and anemia reduce the oxygen-carrying capacity of blood, further aggravating intestinal and hepatic ischemia and forming a vicious cycle of ischemia, bleeding, anemia, and worsening ischemia. This mechanism may explain why the patient presented simultaneously with coffee-ground emesis, severe anemia, and progressive lactate elevation.

### 3.3 Diagnostic challenges of NOMI despite relatively stable blood pressure

Early diagnosis of NOMI remains challenging in emergency and critical care medicine, mainly because its clinical manifestations are non-specific. Although abdominal pain is a common early symptom of mesenteric ischemia (13), pain in NOMI may be absent or disproportionate to the severity of ischemia. Elderly, critically ill, sedated patients, and those with impaired consciousness may lack typical abdominal pain and present only with abdominal distension, gastrointestinal bleeding, or unexplained metabolic disturbances (4, 5). In this case, the early manifestations were mainly coffee-ground emesis, tachypnea, and lethargy, which could easily have been initially attributed to gastrointestinal bleeding or hypovolemia, thereby delaying recognition of NOMI.

The most noteworthy diagnostic pitfall in this case was that apparently stable systemic blood pressure masked severe splanchnic microcirculatory hypoperfusion. At onset, the patient’s blood pressure was 126/68 mmHg; however, he also had tachycardia, altered consciousness, mottled skin, a collapsed inferior vena cava, severe anemia, and marked hyperlactatemia, collectively indicating severe tissue hypoperfusion. These findings highlight that an integrated assessment of hypoperfusion markers, rather than reliance on blood pressure alone, more accurately reflects the true extent of visceral oxygen supply–demand imbalance.

In this case, lactate served as a pivotal early warning biomarker, quantitatively reflecting tissue hypoxia and inadequate perfusion. Previous studies have shown that persistent lactate elevation is closely associated with poor outcomes in critically ill patients, and serum lactate levels above 2 mmol/L are associated with an increased risk of irreversible intestinal ischemia (4, 25). Currently, no specific biomarker can independently confirm the diagnosis of NOMI. Leukocytosis, metabolic acidosis, elevated D-dimer levels, hyperphosphatemia, and hyperamylasemia may all be observed in patients with intestinal ischemia or necrosis, but their specificity is limited (25). Although lactate elevation is not specific for NOMI, it remains an important indicator of tissue hypoxia and splanchnic hypoperfusion (4). In this case, lactate remained persistently elevated and responded poorly to conventional correction of acidosis. Meanwhile, ALT peaked at 1768.60 U/L, further suggesting significant ischemic liver injury. This finding reflects the combined effects of increased intestinal lactate production and reduced hepatic lactate clearance. It should be emphasized that the clinical significance of lactate should not be judged solely by a single absolute value; its dynamic trajectory is equally informative. During the ICU stay, arterial lactate was monitored every 2–4 h, and the results were used in real time to guide the intensity of fluid resuscitation, adjustment of CRRT parameters, and titration of vasoactive agents. Table 1 summarizes arterial blood gas data at key time points, illustrating the overall trends in lactate clearance and restoration of acid–base homeostasis.

Beyond lactate, recent studies have suggested that several gut-derived biomarkers may have potential utility in screening for intestinal ischemia and monitoring ongoing ischemic injury. Intestinal fatty acid-binding protein (I-FABP), which is predominantly expressed in villous epithelial cells of the small intestine, can be rapidly released into the circulation after ischemic injury to the intestinal mucosa and may facilitate the early identification of acute mesenteric ischemia (26–28). D-lactate, a metabolic product of intestinal bacteria, may increase in the setting of intestinal barrier disruption and bacterial translocation (27, 28). Plasma citrulline, a marker of the functional enterocyte mass, has been reported to decrease in association with intestinal mucosal ischemia (28). Other biomarkers, such as α-glutathione S-transferase (α-GST), have also shown potential for the early detection of intestinal ischemia (28). However, the routine availability, optimal diagnostic thresholds, and specificity of these biomarkers for the diagnosis of NOMI remain insufficiently established, and further prospective studies are warranted.

Imaging played a critical role in the management of this case. Contrast-enhanced abdominal CT or computed tomography angiography is currently an important method for identifying NOMI and evaluating the extent of bowel ischemia. Typical findings include bowel wall edema and thickening, decreased or absent bowel wall enhancement, and atherosclerosis of the abdominal aorta and its branches (9, 14). In this case, after the patient’s vital signs stabilized, contrast-enhanced abdominal CT revealed ischemic changes in the bowel and right hepatic lobe, as well as extensive atherosclerosis of the abdominal aorta with mural thrombi, providing direct evidence for the diagnosis of NOMI complicated by IH. Although endoscopy may assist in the diagnosis of ischemic bowel disease, it was not performed because of coagulation abnormalities, potential active gastrointestinal bleeding, and the risk of circulatory instability.

In summary, the early recognition of NOMI currently remains dependent on comprehensive clinical assessment. We therefore recommend a diagnostic strategy centered on serial lactate monitoring and trend analysis, multidimensional assessment of tissue perfusion, and early contrast-enhanced abdominal CT to secure a timely therapeutic window and improve outcomes in this high-risk patient population.

### 3.4 Therapeutic balance between ischemic progression and bleeding risk

The key therapeutic challenge in this case was the simultaneous management of severe splanchnic hypoperfusion and potential gastrointestinal bleeding. Early treatment of NOMI focuses on promptly restoring effective circulating volume, improving tissue oxygen delivery and splanchnic perfusion, and avoiding further mesenteric vasoconstriction. However, this patient had been receiving long-term DAPT and presented with coffee-ground emesis and a marked decrease in hemoglobin, suggesting a risk of gastrointestinal bleeding. Therefore, adjustment of antiplatelet and anticoagulant therapy requires careful balancing of the risks of ischemic progression and worsening bleeding. Had hypoperfusion not been corrected promptly, intestinal and hepatic ischemia could have been further aggravated.

Given this dilemma, the treatment team adopted a strategy centered on intensive supportive care and dynamic evaluation, including fasting, fluid resuscitation, blood transfusion, correction of acidosis, anti-infective therapy, microcirculatory improvement, and close monitoring of blood counts and coagulation function. Because the patient had a high risk of procedure-related bleeding and possible circulatory instability, gastrointestinal endoscopy was not performed immediately. Instead, based on imaging and laboratory findings, priority was given to stabilizing metabolic status and controlling bleeding risk. As gastrointestinal decompression showed no obvious drainage, stool color turned yellow-brown, and hemoglobin levels stabilized, low-molecular-weight heparin anticoagulation was initiated after multidisciplinary evaluation to balance bleeding and thrombotic risks.

Continuous renal replacement therapy played a multidimensional organ-supportive role during the management of this case. On admission to the ICU, the patient presented with severe lactic acidosis that could not be rapidly corrected by conventional volume resuscitation, blood transfusion, and alkalinization therapy; therefore, continuous venovenous hemodiafiltration (CVVHDF) was initiated. On the one hand, CRRT effectively alleviated metabolic acidosis by correcting electrolyte disturbances. Stabilization of the internal milieu not only reduced the metabolic burden but also indirectly mitigated ongoing ischemia of visceral organs, including the intestine and liver, by improving cardiovascular function and systemic circulatory perfusion (29). On the other hand, intestinal ischemia can trigger the substantial release of proinflammatory cytokines, such as TNF-α and IL-6, thereby aggravating endothelial injury, capillary leakage, and microcirculatory dysfunction. Through convective and diffusive clearance, CRRT can continuously remove small- and medium-sized inflammatory mediators, which may theoretically help attenuate an excessively activated systemic inflammatory response (30). In addition, CRRT enables continuous and precise regulation of fluid balance. While maintaining an adequate, effective circulating volume, it may help avoid tissue edema and reductions in organ perfusion pressure that can result from aggressive fluid resuscitation, thereby providing relatively stable circulatory conditions for the gradual restoration of intestinal and hepatic perfusion (31). On the other hand, intestinal ischemia can trigger the substantial release of proinflammatory cytokines, such as TNF-α and IL-6, thereby aggravating endothelial injury, capillary leakage, and microcirculatory dysfunction. Through convective and diffusive clearance, CRRT can continuously remove small- and medium-sized inflammatory mediators, which may theoretically help attenuate an excessively activated systemic inflammatory response (32). In addition, CRRT enables continuous and precise regulation of fluid balance. While maintaining an adequate, effective circulating volume, it may help avoid tissue edema and reductions in organ perfusion pressure that can result from aggressive fluid resuscitation, thereby providing relatively stable circulatory conditions for the gradual restoration of intestinal and hepatic perfusion (33). It should be clearly acknowledged that CRRT is not an etiologic treatment for NOMI or visceral hypoperfusion. Rather, its principal value lies in correcting severe metabolic derangements, attenuating excessive inflammatory activation, and optimizing volume status and hemodynamic homeostasis, thereby securing a critical time window for the recovery of visceral perfusion and subsequent comprehensive diagnostic and therapeutic decision-making.

## 4 Conclusion

This case highlights the risk of severe splanchnic hypoperfusion in elderly surgical patients with panvascular disease receiving long-term DAPT, particularly after perioperative volume fluctuations. Such hypoperfusion may progress to NOMI, IH, and refractory lactic acidosis. In these high-risk patients, serial lactate monitoring and careful assessment of tissue perfusion are essential. Unexplained lactate elevation, metabolic acidosis, gastrointestinal symptoms, or altered mental status should prompt early consideration of occult visceral ischemia, especially NOMI, even when systemic blood pressure appears temporarily stable. Timely imaging, targeted intensive supportive care, and multidisciplinary collaboration may help achieve early diagnosis, limit ischemic progression, and improve clinical outcomes.

## Data Availability

The raw data supporting the conclusions of this article will be made available by the authors, without undue reservation.
